# *De novo* Transcriptome Assembly of a Chinese Locoweed (*Oxytropis ochrocephala*) Species Provides Insights into Genes Associated with Drought, Salinity, and Cold Tolerance

**DOI:** 10.3389/fpls.2015.01086

**Published:** 2015-12-02

**Authors:** Wei He, Huihui Zhuang, Yanping Fu, Linwei Guo, Bin Guo, Lizhu Guo, Xiuhong Zhang, Yahui Wei

**Affiliations:** ^1^Department of Biology, Northwest UniversityXian, China; ^2^Grassland Station, Agriculture and Animal Husbandry BureauZhongwei, China

**Keywords:** locoweeds, *Oxytropis ochrocephala*, transcriptome, stress, Illumina sequencing

## Abstract

**Background:** Locoweeds (toxic *Oxytropis* and *Astraglus* species), containing the toxic agent swainsonine, pose serious threats to animal husbandry on grasslands in both China and the US. Some locoweeds have evolved adaptations in order to resist various stress conditions such as drought, salt and cold. As a result they replace other plants in their communities and become an ecological problem. Currently very limited genetic information of locoweeds is available and this hinders our understanding in the molecular basis of their environmental plasticity, and the interaction between locoweeds and their symbiotic swainsonine producing endophytes. Next-generation sequencing provides a means of obtaining transcriptomic sequences in a timely manner, which is particularly useful for non-model plants. In this study, we performed transcriptome sequencing of *Oxytropis ochrocephala* plants followed by a *de nove* assembly. Our primary aim was to provide an enriched pool of genetic sequences of an *Oxytropis* sp. for further locoweed research.

**Results:** Transcriptomes of four different *O. ochrocephala* samples, from control (CK) plants, and those that had experienced either drought (20% PEG), salt (150 mM NaCl) or cold (4°C) stress were sequenced using an Illumina Hiseq 2000 platform. From 232,209,506 clean reads 23,220,950,600 (~23 G nucleotides), 182,430 transcripts and 88,942 unigenes were retrieved, with an N50 value of 1237. Differential expression analysis revealed putative genes encoding heat shock proteins (HSPs) and late embryogenesis abundant (LEA) proteins, enzymes in secondary metabolite and plant hormone biosyntheses, and transcription factors which are involved in stress tolerance in *O. ochrocephala*. In order to validate our sequencing results, we further analyzed the expression profiles of nine genes by quantitative real-time PCR. Finally, we discuss the possible mechanism of *O. ochrocephala*'s adaptations to stress environment.

**Conclusion:** Our transcriptome sequencing data present useful genetic information of a locoweed species. This genetic information will underpin further research in elucidating the environmental acclimation mechanism in locoweeds and the endophyte-plant association.

## Introduction

Toxic *Oxytropis* and *Astragalus* species (Fabaceae), collectively named locoweeds, pose a threat to livestock on grasslands, primarily in the U.S and China (Zhao et al., 2013). Locoweeds contain the indolizidine alkaloid swainsonine, an α-mannosidase inhibitor which causes over-accumulation of mannose rich oligosaccharide in the lysosomes which impairs the neural system of livestock (Tulsiani et al., 1988). The symptoms of affected animals include staggering, reduced browsing and water consumption, and even death (Ralphs et al., 2002). Locoweed consumption results in a deterioration of animal health and, as a consequence of weight loss, a reduction in meat productive rate. In addition to damaging livestock, the spread of locoweeds destabilizes grassland plant communities and undermines sustainable grassland management. In both the U.S and China, significant annual economic loss has been reported, in some cases up to 20 million USD per year (Creamer and Baucom, 2013; Zhao et al., 2013).

In China, locoweeds are widely distributed and often grow beyond temperate areas, where they encounter reduced rainfall, low temperature and high soil salinity. For example, *O. ochrocephala*, one of the most widely distributed locoweeds in China, is abundant on the grasslands of Qinghai, Gansu, Ningxia and Sichuan Provinces (Zhao et al., 2013), where it occurs in mountains, alpine meadows, open grasslands, and valleys. *O. ochrocephala*'s tolerance to environmental stresses contributes to it rapidly replacing local forage grass species in natural grassland plant communities [Wei He, personal observation]. Plants are often exposed to stressful environment, and have evolved to either avoid or tolerate these abiotic stress, by altering their gene expression, signal transduction, and cellular metabolism. In particular, the underlying gene expression changes trigger accumulation of protective proteins such as heat shock proteins (HSPs) (Wang et al., 2004)and late embryogenesis abundant (LEA) protein (Hundertmark and Hincha, 2008), and regulatory proteins, such as key enzymes in phytohormones synthesis (e.g., abscisic acid and ethylene; Peleg and Blumwald, 2011) and transcription factors that in turn regulate downstream stress inducible genes (Yamaguchi-Shinozaki and Shinozaki, 2006). Currently, the adaptation mechanisms of locoweeds that enable these plants to cope with environmental stress are still ambiguous.

On the other hand, although it has been proved that the production of swainsonine in locoweeds can be attributed primarily to their endophytes (Gardner et al., 2001; Ralphs et al., 2008; Pryor et al., 2009), it is unclear how the intrinsic properties of the plants contribute to the production of locoweed swainsonine. Oldrup et al. (2010) demonstrated in *O. sericea* plants subjected to certain stress conditions, such as drought and low pH, dry mass of its endophyte *U. oxytropis* declines, contrasting to elevated plant biomass and swainsonine concentration. Cook et al. (2013) observed that interactions between endophytes and locoweeds does offer the endophytes nutrients, hormones and some other signals in influencing their capacity to produce swainsonine. These studies imply that locoweeds, as the host of the endophytes, have a direct impact on the production of swainsonine, but the interaction between locoweeds and their symbiotic endophytes remains unclear and needs further investigation.

To resolve these problems, in-depth research at the genetic level is necessary. However, to date limited genetic information of locoweeds is available from public databases (Chung et al., 2004; Archambault and Strömvik, 2011). Although, an EST dataset of a suppressive subtraction cDNA library enriched in genes from two temperate *Oxytropis* species has recently been made available from the NCBI, this only has 1245 ESTs and thus there is a need for further genetic information work in *Oxytropis*. Recent advances in sequencing technology, a reduction in costs, and the development of more data handling programs have resulted in more efforts in genome or transcriptome sequencing as a means of obtaining useful genetic profiles (Dugas et al., 2011; Wei et al., 2011; Liu et al., 2012; Zhou et al., 2012; Gross et al., 2013; Chen et al., 2014). In this study, we analyzed the transcriptomes of *O. ochrocephala* under drought, salt and cold stress. Our primary aim was to provide an enriched pool of genetic sequences of an undescribed *Oxytropis* species for further locoweed research. We use the data obtained to further discuss the possible mechanism of *O. ochrocephala*'s adaptations to stress environmental conditions. The sequences obtained in this study will underpin further research in elucidating the environmental acclimation mechanisms in locoweeds, and in serving as a reference transcriptome to advance the study of endophyte-plant association.

## Materials and methods

### Plant material and RNA extraction

Mature *O. ochrocephala* seeds were collected from Haiyuan, Ningxia Province (N 36°26′49.80,″ E 105°37′26.46,″ altitude: 2656) in July 2013. Seeds were scarified with sandpaper and then imbibed in deionized water for 12 h. All seeds were then placed onto wet filter papers in Petri dishes for germination. Germinated seeds were allowed to grow until the cotyledons emerged. Each seedling was then transferred into an individual pot (5 × 5 × 6 cm) containing a mixture of sand and peat (1:1), and placed into a growth chamber under controlled conditions (photo flux density of 300 μmol m^−2^s^−1^, 14/10 h day/night period; relative humidity of 55–60%; temperature of 25 ± 2°C). All plants were watered weekly until 1 week before stress treatments.

A total of 120 robust 6-week old seedlings were selected and randomized evenly into four groups: (1) control, (2) drought, (3) cold, (4) salt. For the control and cold treatment, plants were watered as normal except that for the cold treatment 10 plants were transferred into a different growth chamber pre-cooled to 4°C. For the drought and salt treatments, 20% PEG-6000 (w/v, polyethylene glycol, Sangon, China) or 150 mM NaCl (Sangon, China) solutions were applied to irrigate the plants in every 4 h, respectively. The rest of growing conditions were as previously described above. A preliminary experiment was set up to test the effect of the stress treatments to the seedlings. Relative electrolyte leakage, malonaldehyde proline content (MDA), proline content, and superoxide dismutase (SOD) and peroxidase (POD) activity were determined at 0, 6, 12, and 24 h post-treatment. Relative water content (RWC) and survival rate were determined 4 d after the treatments. Results showed that all the three stress treatments altered the physiological performance of the plants, and therefore demonstrated that they are effective in the laboratory in mimicking drought, salinity and cold stress in the field (Supplementary Figure 1 in Data Sheet 1).

Plants materials were collected from all individuals in the four groups at 3, 6, and 12 h after the treatments. The three time-points were selected considering that changes at the transcriptomic level are much more rapid than those at the physiological level, as suggested by previous study in Arabidopsis (Kreps et al., 2002). Each sample consisted of 10 seedlings to ensure adequate RNA for RNA-seq. Each whole seedling was removed from the soil, washed and dried, and then immersed in liquid nitrogen immediately. RNA was extracted according to the method of Gasic et al. (2004). To eliminate DNA contamination, total RNA was DNase treated and purified according to the protocol of the manufacturer (Ambion, USA). The integrity of all RNA samples was assessed using 1% denatured agrose gel electrophoresis. RNA purity and concentration were both determined on a NanoDrop ND-1000 Spectrophotometer (NanoDropTechnologies, USA).

### Library preparation and sequencing

A total of 3 μg RNA was prepared from pooling an equal amount of the three post-treatment RNA samples (3, 6, and 12 h for each treatment). Strand non-specific sequencing libraries were generated using NEBNext® Ultra™ RNA Library Prep Kit for Illumina (NEB, USA) following the instructions of the manufacturer. Four index codes were added as indicators for sequences from each sample. Briefly, mRNA was purified from total RNA using oligo-dT attached magnetic beads. Fragmentation was carried out using divalent cations under elevated temperature. First strand cDNA was reverse transcribed using random hexamer primers, with second strand cDNA synthesized subsequently. Remaining overhangs were blunted via exonuclease/polymerase activities. After adenylation of 3′ ends of DNA fragments, NEBNext Adaptor with hairpin loop structure were ligated to prepare for hybridization. In order to select cDNA fragments with a preference of 150~200 bp in length, the library fragments were purified with the AMPure XP system (Beckman Coulter, USA). Then 3 μl USER Enzyme (NEB) was used with size-selected, adaptor-ligated cDNA at 37°C for 15 min followed by 5 min at 95°C before PCR. PCR was performed with Phusion High-Fidelity DNA polymerase, Universal PCR primers and Index Primer. Finally, PCR products were purified with the AMPure XP system (Beckman Coulter, USA). Library quality was assessed with the Agilent Bioanalyzer 2100 system (Agilent Technologies, USA).

The clustering of the index-coded samples was performed on a cBot Cluster Generation System using TruSeq PE Cluster Kit v3-cBot-HS (Illumina, USA) according to the instructions of the manufacturer. After cluster generation, the library preparations were sequenced on an Illumina Hiseq 2000 platform (Illumina, USA) and paired-end reads were generated.

### Sequence annotation

Raw data (raw reads, NCBI Accession: PRJNA292613) were initially processed to generate clean data (clean reads). At this step Q20, Q30, GC-content and sequence duplication levels of the clean data were calculated. *De novo* assembly of the transcriptome was accomplished using Trinity version r20140413p1 (Grabherr et al., 2011) using the Butterfly option, with all parameters set to default except for min_kmer_cov set as 2. Gene function and classification was annotated based on the following databases: Nr, Nt (NCBI non-redundant nucleotide sequences), GO (Gene Ontology), Swiss-Prot, Pfam (Protein family), KOG (euKaryotic Ortholog Groups) and KEGG (Kyoto Encyclopedia of Genes and Genomes).

NCBI blast version 2.2.31+ was employed for annotation against Nr, Nt, SwissProt and KOG databases. The cut-off value was set to 1e^−5^ for Nr, Nt and Swiss-Prot, and 1e^−3^ for KOG. In order to calculate the percent length coverage of unigenes, NCBI blast 2.2.31+ was also used to determine the number of unique top matching proteins that align across more than X% of the lengths of the unigenes (http://trinityrnaseq.sourceforge.net/analysis/full_length_transcript_analysis.html). Pfam annotation was retrieved using HMMER 3.0 package (Finn et al., 2011), with a cut-off value of 0.01. The Nr and Pfam hits were imported into Blast2GO v2.5 for GO classification (Götz et al., 2008), with a cut-off value of 1e^−6^. KEGG annotation was obtained from the KEGG Automatic Annotation Server (Kanehisa et al., 2008). For species similarity analysis, species hits in Nr annotation with the lowest *e*-values were summarized and the results integrated.

### Differential expression analysis

We used the RSEM package version 1.2.0 (Li and Dewey, 2011) to analyze the read counts, which were then converted to FPKM (Fragments Per Kilobase of transcript per Millions fragments sequenced), a commonly accepted estimate for the expression level of unigenes (Trapnell et al., 2010). Prior to differential gene expression analysis, for each sequenced library, we adjusted the read counts with the edgeR program package version 3.0.8 (Robinson et al., 2010) through one scaling normalized factor. Differential expression analysis between the control and each of the three treatment samples was performed using the edgeR package with BCV (Biological coefficient of variation) set as 0.2 (dispersion = 0.04) for exactTest and other parameters using default settings. The threshold for significantly differential expression was set as *q* ≤ 0.005 and |log2 (fold change)|≥1.

### GO enrichment and KEGG pathway enrichment analysis

We performed GO enrichment analysis of the differentially expressed genes (DEGs) was implemented using the GOseq R packages version 1.10.0 based on the Wallenius non-central hyper-geometric distribution (Young et al., 2010), which can adjust for gene length bias in DEGs. KEGG is a database resource for understanding high-level functions and utilities of the biological system, such as the cell, the organism and the ecosystem, from molecular-level information, especially large-scale molecular datasets generated by genome sequencing and other high-throughput experimental technologies (http://www.genome.jp/kegg/). We used KOBAS software version 2.0 (Mao et al., 2005) to test the statistical enrichment of differential expression genes in KEGG pathways (corrected *P* < 0.05).

### Quantitative real-time PCR determination of gene expression

We performed stress treatments and RNA extraction as described above. For cDNA synthesis, 2.5 μg of total RNA was used for reverse transcription with random hexmers according to the instructions of the manufacturer (Thermo Scientific, USA). The cDNA was then diluted 30-fold with nuclease-free water for qRT-PCR. qRT-PCR was performed using a Bio-Rad CFX96 Real-Time PCR system (Bio-Rad, USA). The expression levels of nine genes involved in stress responses were determined (primer sequences, as well as for housekeeping genes were listed in Supplementary Table 1 in Data Sheet 1). The qRT-PCR reactions were performed using the FastStart Universal SYBR GreenMaster system according to the instructions of the manufacturer (Roche, Germany). The qRT-PCR condition was set as recommended by the manufacturer: 95°C for 10 min, 40 cycles at 95°C for 30 s, 58°C for 30 s, and 72°C for 30 s with a dissolving curve followed. Three biological replications and three technical replications, respectively, were used. Relative expression levels were normalized using the 2^−ΔΔCt^ method (Livak and Schmittgen, 2001).

## Results

### Pair-end sequences and assembly

Transcriptomes of four different *O. ochrocephala* samples, including the control (CK), drought (20% PEG), cold (4°C), and salt (150 mM NaCl) stress were sequenced. A total number of 232,209,506 clean reads were generated from sequence reads of the four transcriptomes (NCBI Accession: PRJNA292613), corresponding to 23,220,950,600 (23 G) nucleotides (nt) (Supplementary Table 2 in Data Sheet 1). The average Q20 and Q30 values are 97.61 and 93.06%, respectively; the average GC content is 42.8% (Supplementary Table 2 in Data Sheet 1).

A *de novo* assembly was applied employing all the transcripts from the four treatments. After assembly, 217,270 transcripts (238,301,188 nt) and 118,596 unigenes (87,242,224 nt) were retrieved (Table 1, Assembled sequences of all unigenes were included in Data Sheet 2). Because a proportion of the total unigenes were identified to have non-plant origins after a blast search against the Nr database, these unigenes and the corresponding transcripts were removed from the transcript and unigene pools for downstream analyses. After this adjustment, of all 88,942 unigenes the median length is 368 nt, and N50 is 1237. The length distribution of all the transcripts and unigenes showed that as the length of unigenes increase, the number of unigenes decrease (Supplementary Figure 2 in Data Sheet 1); approximately 17,111 (19%) unigenes are longer than 1 kb.

**Table 1:** **Summary of *de novo* assembly of transcriptome sequencing reads without reference genome**.

	**Median length (nt)**	**Max length (nt)**	**N50**	**N90**	**Total number**	**Total nucleotides (nt)**
Transcripts	658 (706)	15,782 (15,782)	1893 (1920)	440 (466)	217,270 (182,430)	238,301,188 (206,141,699)
Unigenes	379 (368)	15,782 (15,782)	1316 (1237)	280 (273)	118,596 (88,942)	87,242,224 (62,980,053)

*nt, nucleotide; N50 (N90), defined as the length for which the collection of all sequences of that length or longer contains at least half (90%) of the sum of the lengths of all sequences. Non-plant transcripts and unigenes were removed from each pool and adjusted numbers were indicated in brackets*.

### Sequence annotation and similarity analysis

Sequence annotation against seven different public nucleotide/protein databases resulted in the successful annotation of 40,059 (45.03%) unigenes in at least one of the databases, while approximately 55% unigenes remain unmapped in any existing database (Table 2). According to Nr annotation (Nr annotation of unigenes were included in Data Sheet 3), 37,363 (42.01%) unigenes were mapped, whist the remaining unmapped unigenes mainly consist of those ones with lengths ≤ 1000 bp (49,464 out of 51,579, 95.91%). We calculated the percent length coverage of unigenes matching translated sequences in Nr database (Table 3). This showed that there are 14,501 (38.8%) unigenes that are represented by nearly full-length transcripts, having >80% alignment coverage.

**Table 2:** **Summary of unigenes annotation against public available databases**.

	**Number of unigenes**	**Percentage (%)**
Annotated in Nr	37,363	42.01
Annotated in Nt	24,328	27.35
Annotated in KO	10,465	11.76
Annotated in SwissProt	21,844	24.55
Annotated in Pfam	24,552	27.60
Annotated in GO	24,983	28.08
Annotated in KOG	11,596	13.03
Annotated in all Databases	5198	5.84
Annotated in at least one Database	40,059	45.03
Total Unigenes	88,942	100

*Nr, NCBI non-redundant protein sequences; Nt, NCBI non-redundant nucleotide sequences; KO, KEGG Ortholog database; Swiss-Prot, A manually annotated and reviewed protein sequence database; Pfam, Protein family; GO, Gene Ontology; KOG/COG, Clusters of Orthologous Groups of proteins*.

**Table 3:** **Distribution of percent length coverage of unigenes matching translated sequences in the Nr database**.

**Coverage**	**Unigene count**	**Percentage (%)**	**Accumulated unigene count**	**Accumulated percentage (%)**
100% ≥ X > 90%	12,233	32.7	12,233	32.7
90% ≥ X > 80%	2268	6.1	14,501	38.8
80% ≥ X > 70%	1917	5.1	16,418	43.9
70% ≥ X > 60%	2136	5.7	18,554	49.6
60% ≥ X > 50%	2195	5.9	20,749	55.4
50% ≥ X > 40%	2359	6.3	23,108	61.7
40% ≥ X > 30%	2844	7.6	25,952	69.3
30% ≥ X > 20%	3747	10.0	29,699	79.3
20% ≥ X > 10%	5026	13.5	34,725	92.8
10% ≥ X > 0%	2638	7.1	37,363	100

**Figure 1:**
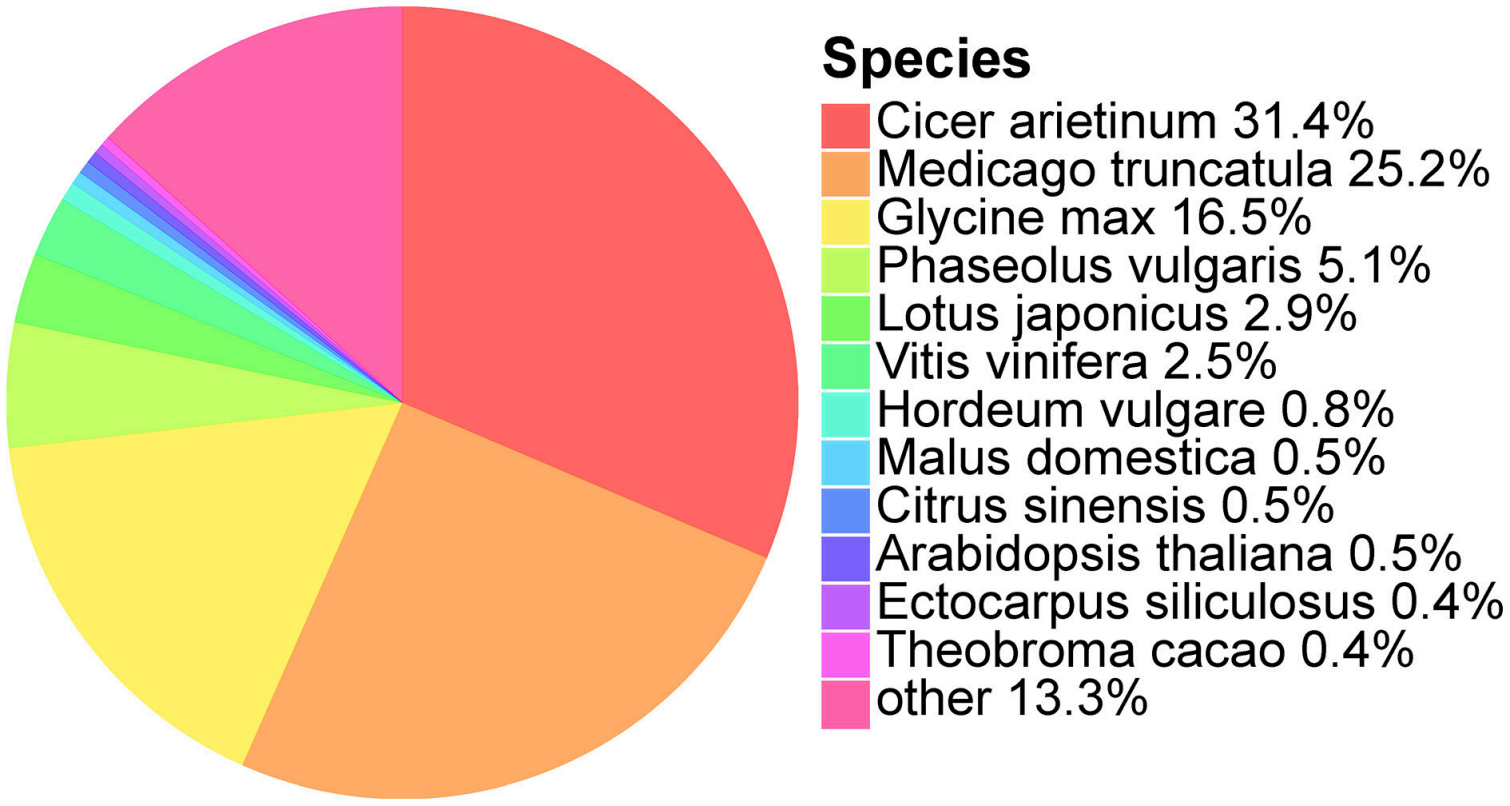
**Species classification based on the best Nr hits of the unigenes identified in the *de novo* assembled transcriptomes**. Percentage of the unigenes which has the best species similarity, to the total number of unigenes, is indicated following a species name.

We further analyzed the annotated unigenes for their organism similarity to other plant species in the NCBI Nr database. Species hits from Nr were selected and the most abundant 12 species are shown (Figure 1). The top five matches are *Cicer arietinum* (31.4%), *Medicago truncutula* (25.2%), *Glycine max* (16.5%), *Phaseolus vulgaris* (5.1%), and *Lotus japonicas* (2.9%). All of these five species belong to the Fabaceae, the same family as *O. ochrocephala*, and explains up to 82.1% of all the annotated unigenes. The remaining unigenes (17.9%) are similar to other 401 plant species.

### Functional classification of the unigenes

**Figure 2:**
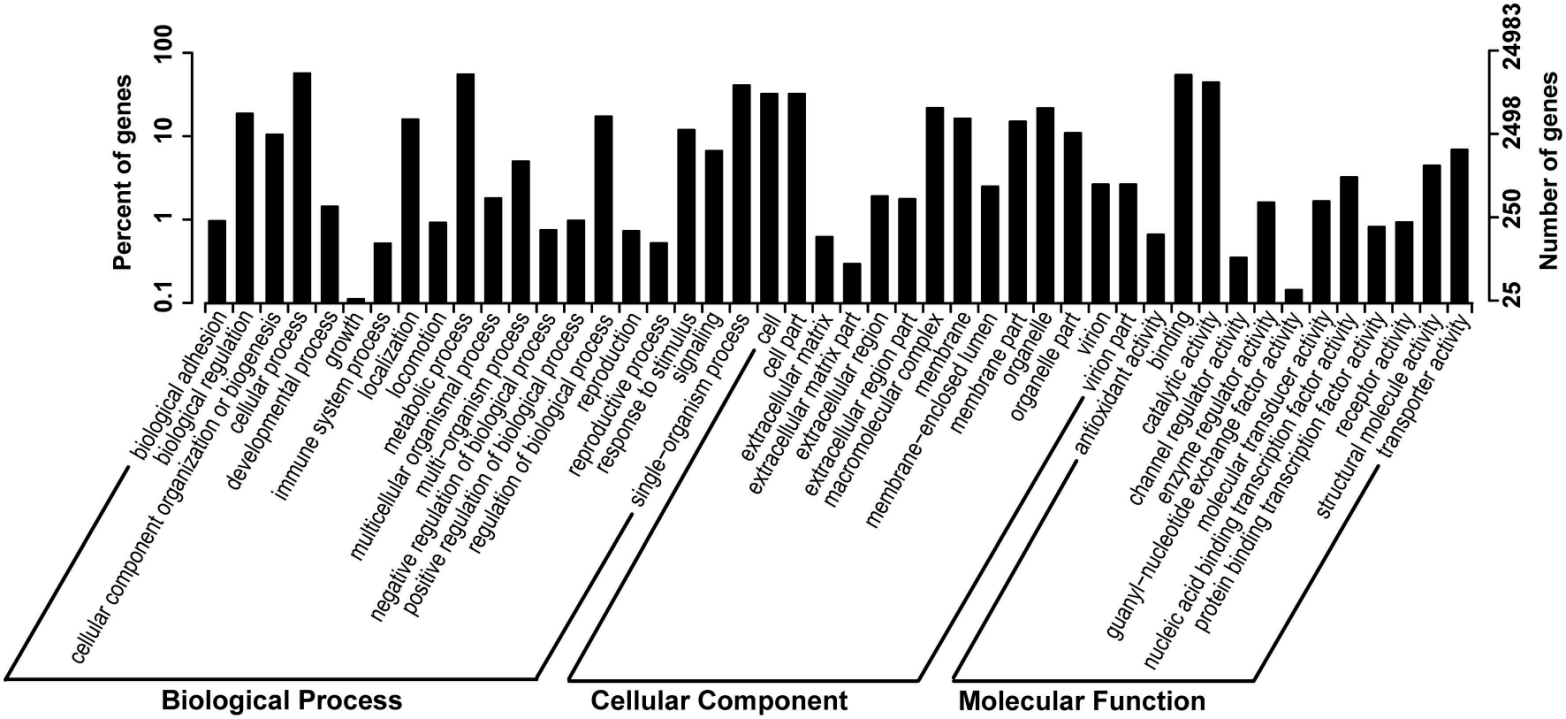
**GO (gene onthology) annotation of the unigenes**. Percentage of genes and number of genes in biological process, cellular component and molecular function are shown, respectively.

In order to study the putative function of the unigenes we identified, we classified all unigenes and their deduced protein into different functional groups, by employing GO and KOG annotation, and KEGG pathway classification. GO annotation is the most widely used classification to group genes by their putative function, and similarly, KOG and KEGG pathway classifications also provided useful information in clustering putative genes or proteins by their functions. We present GO annotation here as an example of gene functional classification (Figure 2). In GO annotation, 24,983 out of 88,942 unigenes (28.08%) are successfully assigned into 46 functional groups, all of which belong to one of the basic three ontologies: biological process, cellular component, and molecular function (Figure 2). The highest proportion of these unigenes is characterized by “cellular process,” “metabolic process,” “binding,” “catalytic process,” “cell and cell part.” KOG and KEGG pathways are shown in Supplementary Figures 3, 4 in Data Sheet 1.

### Differentially expressed genes under stress conditions

**Figure 3:**
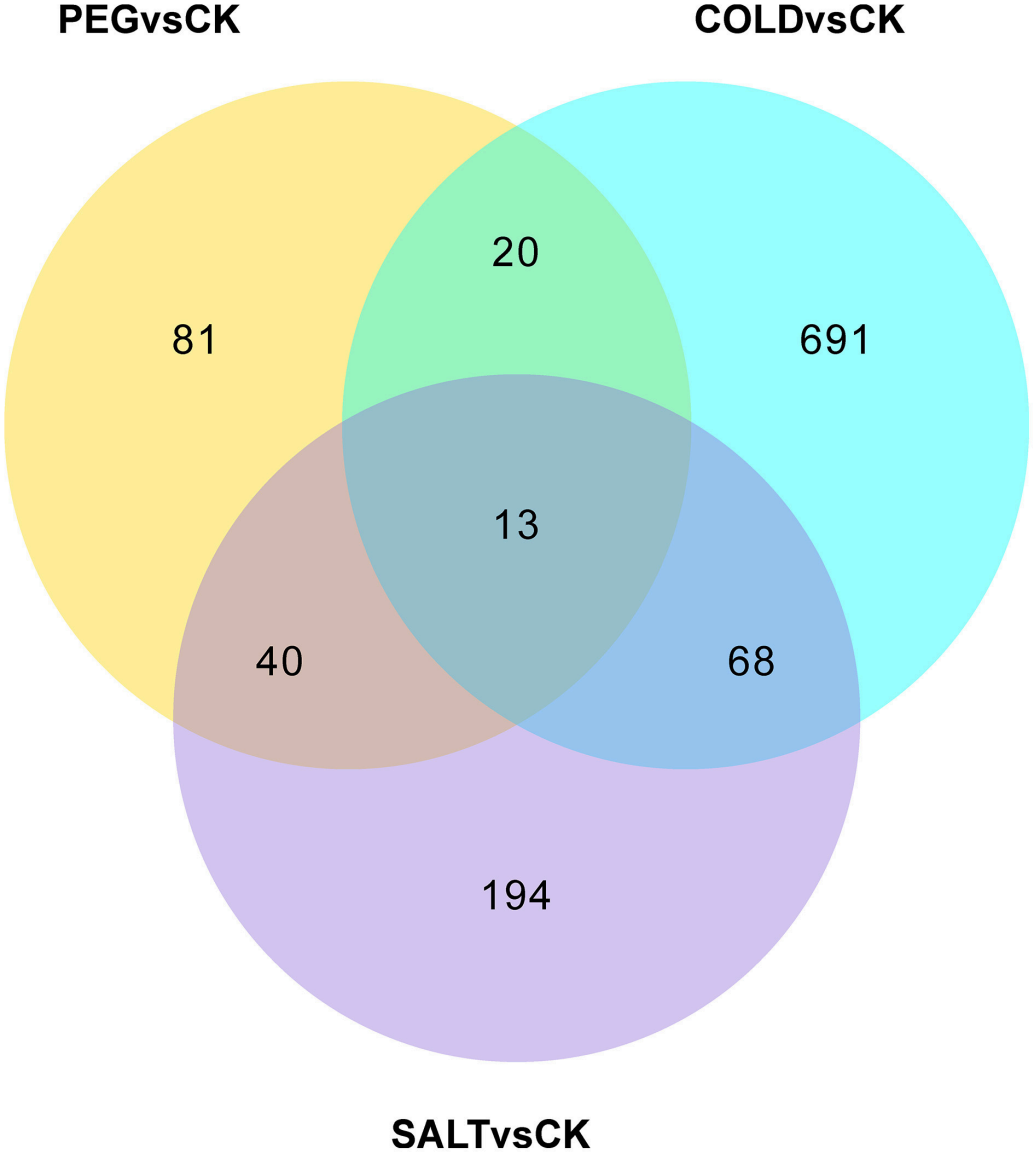
**Venn diagram showing the numbers of genes expressed differentially under the stress treatments and control**.

**Figure 4:**
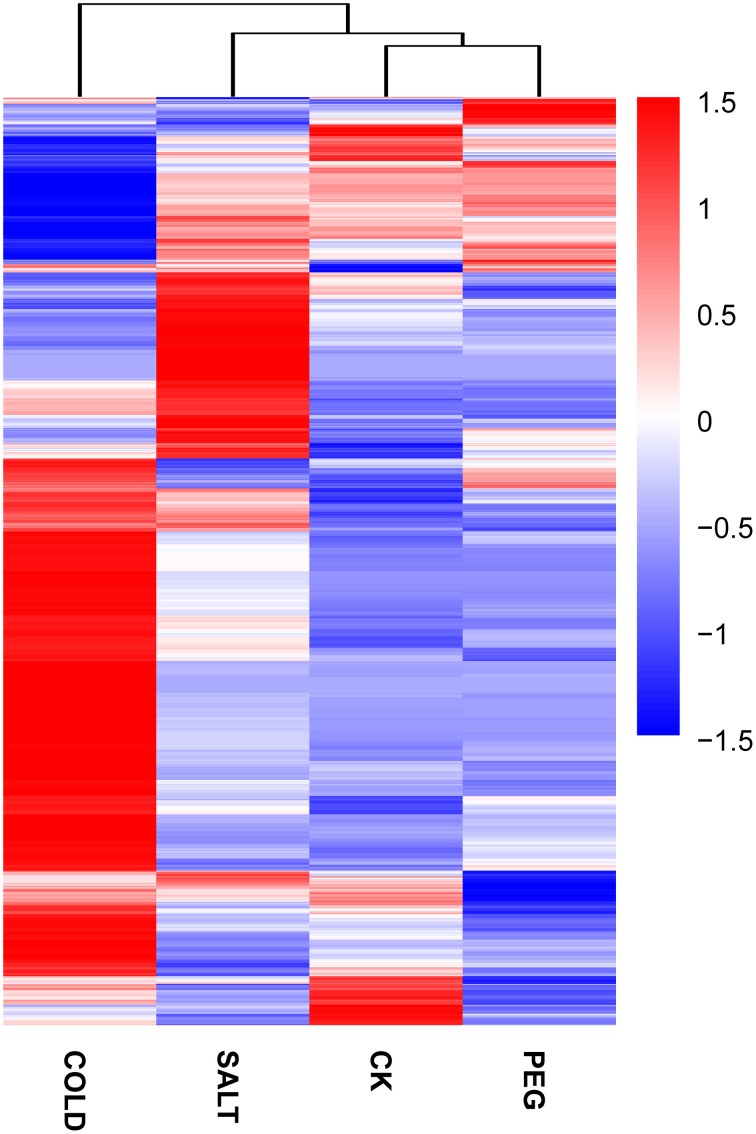
**Expression pattern and clustering of genes expressed differentially under the stress treatments and control**. Blue to red colors reflect gene expression levels indicated as $\log_{10}^{\left(\text{FPKM}+1\right)}$ (value of −1.5–1.5). A detailed heatmap with unigene IDs and annotations was included (Data Sheet 6).

In order to determine the patters of differentially expressed genes (DEGs) in *O. ochrocephala* under drought, salinity and cold stress, normalized read counts of all unigenes and gene expression levels, estimated as FPKM, were determined. This showed distinct expression patterns among the treatments and control (Figures 3, 4). The most distinct gene expression pattern was exhibited by cold stressed plants, followed by those plants that had experienced saline and drought stress. Through, GO and KEGG pathway enrichment, DEGs were further categorized into different functional groups (Table 4, Data Sheets 4, 5). Most of the genes relate to enzymes in plant metabolite biosynthesis pathways or transcription factors, and are up-regulated in the three stress treatments. However, we observed that more genes are involved in cold stress than those in drought and saline stress, especially drought stress.

**Table 4:** **Putative genes involved in stress tolerance in *Oxytropis ochrocephala***.

**Gene ID**	**Annotation/Species**		**Sample**	**log2^(fold change)^**	***q*-value**
**HEAT SHOCK PROTEINS**					
comp25961_c0	Heat shock protein 83 [*Medicago truncatula*]		Cold	−1.09	1.58E-12
comp68481_c0	Heat shock protein 70 [*Ziziphus jujuba*]			−1.80	1.99E-03
comp70341_c0	18.2 kDa class I heat shock protein [*Glycine max*]			−2.14	4.81E-04
comp74708_c0	Small heat shock protein C4 [*Glycine max*]			−4.09	1.61E-03
comp80742_c0	Heat shock protein 81-1 [*Theobroma cacao*]			−2.53	4.63E-03
comp81398_c0	22.7 kDa class IV heat shock protein [*Medicago truncatula*]			−2.69	2.08E-04
comp83116_c0	26.5 kDa heat shock protein [*Medicago truncatula*]			−3.88	3.82E-08
comp83893_c0	18.2 kDa class I heat shock protein [*Cicer arietinum*]			−2.22	1.01E-04
comp85605_c0	Heat shock protein [*Glycine max*]			−3.09	2.75E-08
comp87890_c0	Heat shock protein (hsp17.9) [*Pisum sativum*]			−2.47	2.99E-06
comp90848_c0	18.2 kDa class I heat shock protein [*Medicago truncatula*]			−2.67	1.39E-06
**LEA PROTEINS**					
comp83922_c0	Late embryogenesis abundant domain-containing protein [*Arabidopsis thaliana*]		Drought	−2.99	1.01E-03
comp76465_c0	Late embryongenesis abundant protein [*Medicago truncatula*]			2.97	4.61E-03
comp77463_c0	Y2K4 dehydrin variant G3, partial [*Medicago sativa*]			1.89	4.43E-03
comp77835_c0	Late embryogenesis abundant protein group 9 protein [*Arachis hypogaea*]		Salt	4.74	3.24E-10
comp80885_c0	Late embryogenesis abundant protein-1 [*Cicer arietinum*]			5.92	1.22E-25
comp80885_c1	Late embryogenesis abundant protein-2 [*Caragana jubata*]			3.66	1.76E-07
comp81820_c0	Soybean seed maturation polypeptides [*Glycine max*]			2.98	9.26E-08
comp84138_c0	Late embryogenesis abundant protein [*Medicago truncatula*]			6.34	1.10E-16
comp77463_c0	Y2K4 dehydrin variant G3 [*Eriobotrya japonica*]			4.26	9.51E-43
comp72368_c0	Putative late-embryogenesis protein-like [*Ammopiptanthus mongolicus*]		Cold	2.39	5.17E-06
comp80481_c0	Late embryogenesis abundant protein 1 [*Medicago truncatula*]		Cold	2.53	7.67E-18
comp70033_c0	Late embryogenesis protein-like protein [*Glycine max*]			2.21	1.27E-04
**METABOLITES**					
comp76128_c0	Chalcone reductase 1 [*Astragalus membranaceus*]		Drought	1.31	2.47E-13
comp76729_c0	Isoflavone reductase-like protein [*Medicago truncatula*]		Salt	1.05	9.63E-16
comp83244_c0	6a-hydroxymaackiain methyltransferase [*Pisum sativum*]			1.53	6.67E-04
comp76428_c0	Cinnamate 4-hydroxylase [*Astragalus membranaceus*]			1.12	8.33E-09
comp60088_c0	4-coumarate CoA ligase mRNA, complete cds [*Medicago truncatula*]			1.04	1.30E-05
comp67410_c0	Caffeoyl-CoA O-methyltransferase [*Caragana korshinskii*]			1.07	3.13E-08
comp76128_c0	Chalcone reductase 1 [*Astragalus membranaceus*]			1.42	1.61E-15
comp84899_c0	Isoliquiritigenin 2'-O-methyltransferase [*Medicago truncatula*]			1.40	6.34E-34
comp91892_c0	Phenylalanine ammonia-lyase [*Astragalus mongholicus*]			1.43	3.85E-13
comp91892_c0	Phenylalanine ammonia-lyase [*Astragalus mongholicus*]		Cold	1.82	4.12E-55
comp82145_c0	Phenylalanine ammonia-lyase [*Astragalus mongholicus*]			2.26	3.05E-05
comp87406_c0	Phenylalanine ammonia lyase [*Astragalus membranaceus*]			2.69	8.18E-32
comp81759_c0	Isoflavone 7-O-methyltransferase [*Glycyrrhiza echinata*]			1.71	3.84E-03
comp83244_c0	6a-hydroxymaackiain methyltransferase 1 [*Pisum sativum*]			1.58	3.53E-06
comp91058_c2	Chalcone synthase 1 [*Astragalus membranaceus*]			1.99	3.67E-16
comp86596_c0	Malonyl-CoA:isoflavone 7-O-glucoside-6”-O-malonyltransferase [*Glycine max*]			1.77	3.84E-03
comp82821_c0	Laccase-7-like [*Cicer arietinum*]			1.31	4.35E-04
comp91112_c1	Anthocyanin 3'-O-beta-glucosyltransferase [*Medicago truncatula*]			−1.75	1.84E-30
comp77150_c0	Beta-glucosidase 12-like isoform X3 [*Glycine max*]			2.69	3.68E-04
comp62108_c0	Gibberellin 2-beta-dioxygenase 1 [*Medicago truncatula*]			1.95	4.55E-04
comp80680_c0	Zeaxanthin epoxidase [*Cicer arietinum*]			−1.90	5.72E-04
comp76729_c0	Isoflavone reductase-like protein [*Medicago truncatula*]			1.05	9.63E-16
**HORMONE RELATED**					
comp81166_c0	1-aminocyclopropane-1-carboxylate oxidase [*Cicer arietinum*]	Ethylene	Drought	2.85	4.72E-06
comp73104_c0	Allene oxide cyclase [*Medicago truncatula*]	JA		3.83	1.12E-06
comp86382_c0	Alcohol dehydrogenase [*Medicago truncatula*]	ABA		1.86	4.54E-03
comp84731_c4	PR1-b gene for putative basic PR1 protein [*Cicer arietinum*]	SA		1.58	3.47E-12
comp86683_c0	9-cis-epoxycarotenoid dioxygenase [*Caragana korshinskii*]	ABA	Salt	3.21	3.08E-09
comp80804_c0	Ent-kaurenoic acid hydroxylase [*Medicago truncatula*]	GA	Cold	−1.12	6.90E-14
comp62108_c0	Gibberellin 2-oxidase [*Medicago truncatula*]			1.95	4.55E-04
comp80680_c0	Zeaxanthin epoxidase [*Cicer arietinum*]	ZR		−1.90	5.72E-04
comp87210_c0	Cytokinin-O-glucosyltransferase [*Medicago truncatula*]			1.80	1.46E-03
comp84200_c0	Auxin-induced protein [*Ricinus communis*	IAA		2.35	5.24E-30
comp72368_c0	Indole-3-acetic acid-induced protein [*Glycine max*]			2.39	5.17E-06
comp78994_c0	Abscisic acid 8′-hydroxylase [*Medicago truncatula*]	ABA		−2.18	1.15E-04
comp79621_c0	ABA-inducible protein [*Caragana jubata*]			1.25	9.63E-06
comp82821_c0	Laccase-7-like [*Cicer arietinum*]	Cytokinin		1.31	4.35E-04
**TRANSCRIPTION FACTORS**					
comp87209_c3	Homeodomain leucine zipper protein [*Cicer arietinum*]	bZI	Salt	7.15	2.04E-05
comp67029_c1	bZIP transcription factor [*Caragana korshinskii*]			1.39	1.33E-05
comp72293_c0	Homeobox-leucine zipper protein [*Medicago sativa*]			2.42	9.97E-05
comp81505_c0	Transcription factor bHLH25-like [*Glycine max*]	bHLH		2.56	2.06E-04
comp85725_c0	Transcription factor bHLH25-like [*Glycine max*]			2.08	1.15E-03
comp71824_c0	NAC transcription factor-like protein [*Medicago truncatula*]	NAC		2.29	9.09E-13
comp84372_c0	R2R3-MYB transcription factor [*Medicago truncatula*]	MYB		2.32	5.58E-05
comp76034_c0	WRKY transcription factor 33 [*Cicer arietinum*]	WRK	cold	2.07	1.39E-04
comp82399_c1	WRKY transcription factor 40-like isoform 1 [*Glycine max*]	WRKY		1.83	1.02E-03
comp87093_c1	WRKY transcription factor 41 [*Cicer arietinum*]	WRK	Cold	1.78	1.96E-03
comp69006_c0	ER transcription factor 1B-like [*Glycine max*]	ER-TF		2.97	5.50E-09
comp70325_c0	ER transcription factor ERF061 [*Medicago truncatula*]	ER-TF		2.38	3.23E-03
comp77817_c0	ER transcription factor 11 [*Medicago sativa*]	ER-TF		1.13	1.58E-06
comp83387_c0	ER transcription factor [*Cicer arietinum*]	ER-TF		1.36	4.69E-05
comp86762_c0	ER transcription factor 7-like [*Cicer arietinum*]	ER-TF		1.40	9.05E-07
comp90492_c1	ER transcription factor ERF023-like [*Cicer arietinum*]	ER-TF		−2.01	3.78E-03
comp70325_c0	ER transcription factor ERF061 [*Medicago truncatula*]	ER-TF		2.37	1.47E-05
comp89496_c2	ER transcription factor ERF098-like [*Glycine max*]	ER-TF		2.72	2.96E-05
comp90492_c1	ER transcription factor ERF023-like [*Cicer arietinum*]	ER-TF		−2.01	3.57E-04
comp71742_c1	AP2/ERF and B3 domain-containing transcription repressor [*Cicer arietinum*]	ER-TF		2.18	5.51E-04
comp56812_c1	ER transcription factor 1B-like [*Cicer arietinum*]	ER-TF		3.75	9.79E-04
comp23350_c0	bZIP transcription factor [*Glycine m*	bZIP		1.20	3.99E-09
comp85721_c0	bZIP transcription factor bZIP105 [*Glycine max*]			1.39	1.51E-07
comp87209_c3	Homeobox-leucine zipper protein ATHB-40 [*Cicer arietinum*]			7.22	5.28E-06
comp69091_c0	Glycine max bZIP transcription factor bZIP35 [*Glycine max*]			2.10	2.10E-04
comp85114_c0	Transcription factor bHLH25 [*Medicago truncatula*]	bHLH		−1.62	4.87E-03
comp83156_c0	Transcription factors MYC4 [*Medicago truncatula*]			3.00	1.67E-06
comp91054_c0	Transcription factor bHLH96 [*Cicer arietinum*]			−1.87	2.92E-03
comp90383_c1	NAC domain protein NAC2 [*Cicer arietinum*]	NAC		1.71	2.92E-03
comp83816_c0	NAC domain protein NAC1 [*Glycine max*]			2.46	5.91E-05
comp71824_c0	NAC domain protein [*Medicago truncatula*]			2.21	3.36E-05
comp82153_c0	MYB transcription factor [*Medicago truncatula*]	MYB		4.52	4.07E-18
comp80312_c0	MYB family transcription factor-like protein [*Medicago truncatula*]			2.19	4.83E-03

**Figure 5:**
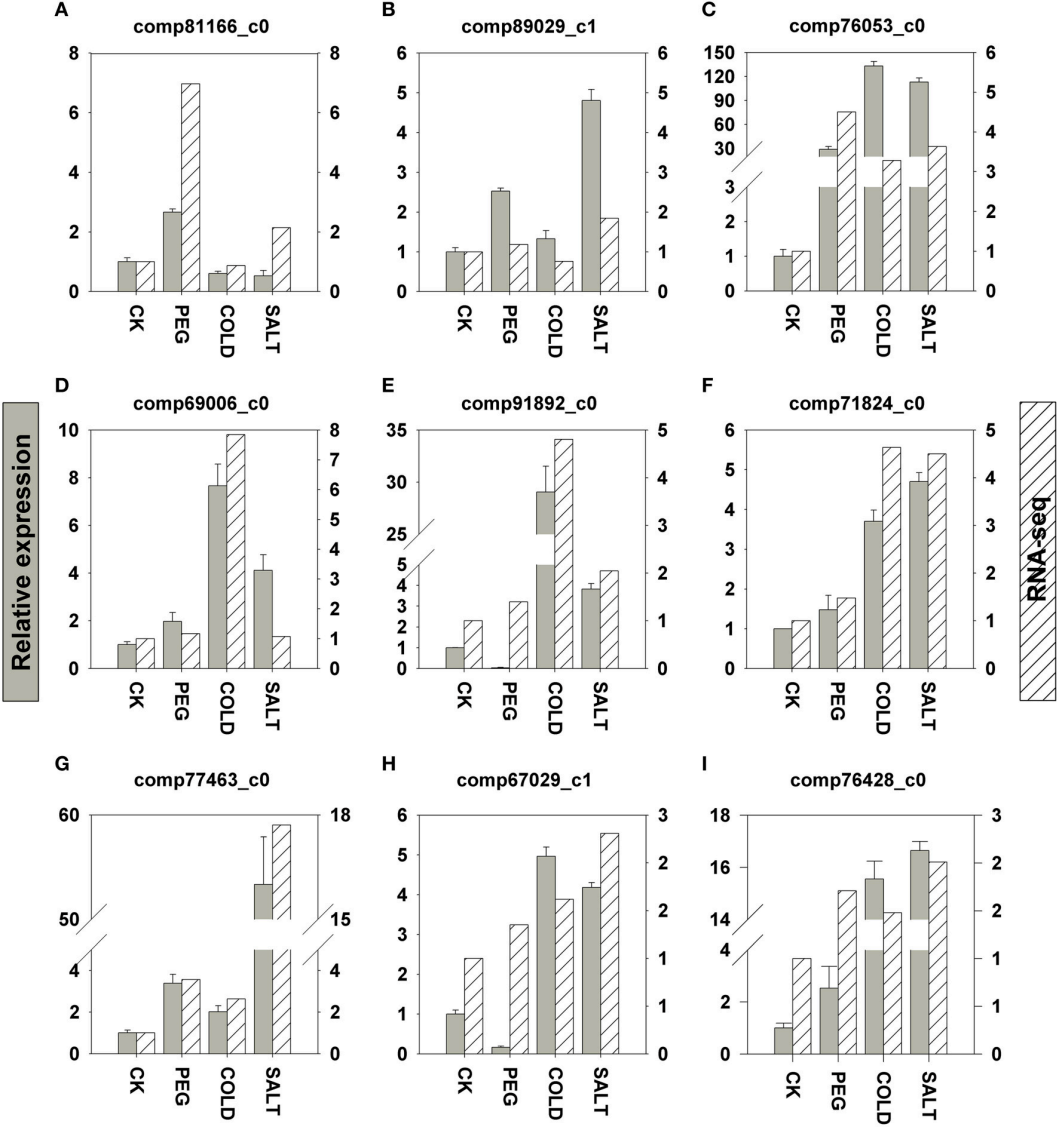
**qRT-PCR validation of nine DEGs related to drought (A–C), cold (D–F), and saline (G–I)**. Y axes in the graphs show normalized fold changes in qRT-PCR (left) and FPKM in RNA-seq (right). qRT-PCR expression levels were normalized against *ACTIN* and *HISTONE*. Error bars represent one standard error of the mean (*n* = 3). CK: control sample. **(A)** comp81166_c0: 1-aminocyclopropane-1-carboxylate oxidase. **(B)** comp89029_c1: galactinol synthase. **(C)** comp76053_c0: peroxidase, **(D)** comp69006_c0: ethylene-responsive transcription factor. **(E)** comp91892_c0: phenylalanine ammonia-lyase. **(F)** comp71824_c0: NAC transcription factor. **(G)** comp77463_c0: Y2K4 dehydrin variant G3. **(H)** comp67029_c1: bZIP transcription factor. **(I)** comp76428_c0: cinnamate 4-hydroxylase.

In order to validate the transcriptomic sequencing results from DEGs, we further performed qRT-PCR (quantitative Real-Time PCR) analyses on nine genes involved in the three stress responses (Figure 5). These genes were selected based on the fact that either their expression patterns change remarkably according to the DEG data or their functions had been well established in previous studies in plant stress responses to drought, salinity and cold. All the genes we tested are up-regulated in response to the three stress treatments, respectively. In general, the differential expression profiles from the qRT-PCR results showed similar patterns to FPKM fold change. Nevertheless, the expression fold changes in four genes are more drastic in the qRT-PCR results than those in the DEGs.

## Discussion

### Sequencing and annotation

In this study we performed RNA sequencing and report a *de novo* assembly of a common locoweed species in China, *O. ochrocephala*. In total, 88,942 unigenes with an N50 of 1237 were obtained, of which 19% are longer than 1 kb. Furthermore, of the 37,363 unigenes annotated to Nr, 38.8% are near full-length sequences matching >80% of the matched protein sequences. Our data are comparable to many other transcriptome studies (Garg et al., 2011; Xie et al., 2012; Fu et al., 2013; Farrell et al., 2014; Ge et al., 2014), and indicate satisfactory sequencing and assembly quality. Nevertheless, those un-annotated unigenes demonstrate that in *Oxytropis* spp, sequencing depth has not yet been fully reached, and some of these sequences may have functions specific to the genus.

It is interesting that in GO annotation, very few unigenes were observed within the term “growth.” Indeed, seedlings of *O. ochrocephala* in this study had reduced growth rates when established from seeds and subsequently grew slowly in a greenhouse. Different attempts were made to change the growth conditions by altering the combination of light, temperature, moisture, and seasonal time of planting, and by using seed accessions from various sites in China. None of these trials produced optimal seedling growth. In addition, we have observed that *O. ochrocephala* plants grown on grasslands with intensive grazing have reduced plant height than the plants in a reserve park in Ningxia Province with lower grazing pressure. Moreover, when field collected seeds develop into seedlings in the laboratory, Ningxia accession always outperformed Qinghai accession (data not shown). We suggest that the slow growth rate of *O. ochrocephala* is likely to be attributed to its life cycle strategy, in which the syntheses of toxins and antinutrients are trade-off to growth. As defensive compounds, secondary metabolites are costly in nature and their production may occur at the expense of plants' growth (Vincent et al., 2005; Treutter, 2006). Ecological theory predicts that plants may invest less energy and resources into growth than into accumulating defensive metabolites as a strategy to deter herbivores, to subsequently outcompete faster growing intra- and/or inter-specific individuals (Blumenthal, 2006; Fine et al., 2006).

### Differential expression patterns of stress response genes by sequencing

DEG analysis revealed genes expressed differentially under drought, cold, and saline stress conditions in *O. ochrocephala*. There are more genes involved in cold stress response that those in drought and saline stress, suggesting that *O. ochrocephala* plants are not as sensitive to drought and saline stress as they are to cold stress. These genes in different functional groups are highly likely to be involved in stress response in *O. ochrocephala*. Firstly, the expression levels of protective proteins are altered, such as HSP and LEA. HSPs are a group of stress-induced proteins, which have been shown to act as a chaperone of proteins that regulate growth and development, thus preventing proteins from aggregating and denaturing (Kotak et al., 2004; Nishizawa et al., 2006). We identified 11 HSP unigenes in *O. ochrocephala*. One unigene is closely related to HSP83, one to HSP81, one to HSP70, and the others are annotated as either “small heat shock protein” or “heat shock factors.” These unigenes have top BLAST hits against sequences from legumes such as *Cicer arietinum, Medicago truncatula*, and *Glycine max*, to non-legumes such as *Ziziphus jujube*. In addition, three unigenes, with the highest similarity to sequences in *Medicago truncatula, Medicago sativa*, and *Glycine max*, respectively, were all annotated to an 18.2 kDa class I heat shock protein. These results suggest that there may be a diversity of HSP genes in *O. ochrocephala*. In this study, DEGs of all HSPs were only found in the cold treatment and they are down-regulated by on average 6.1-fold. This is in contrast to many studies, in which HSPs usually show changes with both increase and decrease (Hu et al., 2009). However, no transcripts of HSPs were found with significantly differential expression profiles in the drought and saline treatments, probably indicating that the expression of HSPs in these two treatments are not drastically altered.

LEA proteins are ubiquitous proteins that are highly stress inducible. LEA proteins are rapidly synthesized in those vegetative tissues experiencing water deficiency and seeds in desiccation in both desiccation insensitive and resurrection plants (Hundertmark and Hincha, 2008). LEA proteins may prevent molecular denaturation, by facilitating hydration in target proteins and other macromolecules (Wang et al., 2004). Several functional studies have confirmed a protective role of LEA proteins and dehydrins against drought and osmotic stress tolerance (NDong et al., 2002). In *O. ochrocephala*, many LEA genes express abundant transcripts in the three abiotic stresses, and except one unigene, all the others are up-regulated.

Secondly, as regulatory proteins, key enzymes in phytohormones synthesis and transcription factors are up-regulated by the three stress treatments. Secondary metabolites possess diverse protective or signaling functions in plant defense. In addition, they help certain plants gain completive edge over the others in a community through a mechanism known as allelopathy (Hierro and Callaway, 2003). In our transcriptome dataset, DEGs were identified in the biosynthesis pathway of flavonoids, alkaloid, phenolics, lignin, coumarins, anthocyanins, cyanogenic glycosides, terpenoids, and steroidal compounds (Table 4). Many of the DEGs are in the flavonoid pathway, one of the best-characterized metabolic pathways in the secondary metabolism in plants (Treutter, 2006). Previous studies suggested that certain flavonoids play a key role in mediating the ROS (reactive oxygen species) homeostasis in plants as scavengers, and therefore, enhance plants' tolerance abiotic stress. In our study, several unigenes encoding key enzymes in the flavonoid biosynthesis pathway showed differential expression patterns in stress conditions, including phenylalanine ammonia lyase (comp91892_c0, comp82145_c0, and comp87406_c0), cinnamate 4-hydroxylase (comp76428_c0), 4-coumarate CoA ligase (comp60088_c0), chalcone synthase (comp91058_c2) and chalcone reductase (comp76128_c0). The differential expression patterns of the genes involved in flavonoid synthesis suggest that flavonoids may be active in the *O. ochrocephala* response to environmental stress. This result agrees with previous studies that have found these enzymes are up-regulated in stressed plants (Sallaud et al., 1995; Treutter, 2006; Gao et al., 2008).

Plant hormones are known to be involved in plant responses to various stresses (Shinozaki and Yamaguchi-Shinozaki, 2006). For instance, abscisic acid (ABA) is essential for various stress responses and the endogenous ABA level changes drastically in ABA dependent stress responses. Genes upstream of and in the ABA synthesis pathway can be up-regulated under water deficiency (Nambara and Marion-Poll, 2005). We found that some key enzymes in ABA biosynthesis such as 9-cisepoxycarotenoid dioxygenase (NCED) (comp86683_c0) and alcohol dehydrogenase (comp86382_c0) were both up-regulated and 8′-hydroxylase (comp78994_c0), the enzyme for the oxidative catabolism of ABA, was down-regulated under high salinity conditions. This confirms the role of ABA in regulating the transcriptional profile in *O. ochrocephala*. Bioactive gibberellins (GAs) control diverse aspects of growth and development, including seed germination, stem elongation, leaf expansion, and flower and seed development (Richards et al., 2001). We found that one unigene (comp80804_c0) encoding ent-kaurenoic acid oxidase, the key enzyme in GA biosynthesis, was down-regulated by exposure to cold, whilst a unigene encoding GA 2-oxidase (comp62108_c0) in the GA deactivation pathway was up-regulated. Both processes may result in a decreased endogenous level of bioactive GA, similar to other plant species (Achard et al., 2008). Cytokinin activity promotes cell expansion through cell wall loosening (Thomas et al., 1981). The control of cytokinin levels may be an important mechanism through which cell expansion is controlled in *O. ochrocephala*. Our data suggested that cytokinin degradation in the apoplast may be affected by exposure to cold. A cytokinin-O-glucosyltransferase gene (comp87210_c0) was specifically up-regulated in the cold and saltine treatments, along with a laccase gene (comp82821_c0). The former enzyme reversibly converts cytokinins to ortho-glucosyl derivatives, and temporarily reduces cytokinin activity (Martin et al., 1999). The latter enzyme has been proposed to function together in the apoplast to degrade cytokinins (Galuszka et al., 2005). Taken together, changes in plant hormone levels inevitably influence the production of secondary metabolites, and it is conceivable that cross-talk among these hormones as well as other signal molecules function together to endow *O. ochrocephala* plants with resistance to stress.

Transcription factors also respond to stress signals at the early stage during stress. First, the WRKY transcription factors, which may be involved in the initial steps of the defense-response signaling pathway, often act as repressors as well as activators for transcription, and members of this family function in abiotic stresses such as drought and cold (Rushton et al., 2010; Tripathi et al., 2013). In *O. ochrocephala*, unigenes (comp76034_c0 and comp87093_c1) annotated as WRKY transcription factors are up-regulated during cold exposure. Second, we found a total of 12 ethylene-responsive (ER) transcription factor genes belonging to the AP2/EREBP family to be a response to cold in *O. ochrocephala*. Ethylene-responsive (ER) transcription factors, which are related to the C-repeat binding transcription factors (CBFs), have been observed to be expressed in plants in response to abiotic stresses (Lee et al., 2007). For example, CBFs were identified as a key responder to low temperature stress (Zhou et al., 2011). In the CBF dependent pathway, the CRT/DRE cis-element in *COR* (cold response genes) is recognized by the CBFs, and as a result, the downstream genes are activated. This results in chilling and freezing tolerance in plants (Gilmour et al., 1998). In *O. ochrocephala*, we identified two genes (comp22730_c0 and comp85560_c1) homologous to *COR*. In Arabidopsis, *CBF1* encodes an AP2 domain containing a transcriptional activator that binds to the C-repeat/DRE in response to low temperature and water deficiency (Gilmour et al., 2000; Haake et al., 2002). However, the CBF genes and their targets COR genes in *O. ochrocephala* are induced by cold stress only, not by drought. This suggests that there may be different mechanisms of dehydration responses in *O. ochrocephala* and Arabidopsis. Third, in this study, we found that three basic leucine zipper (bZIP) family genes encoding AREB (ABA-responsive element binding) and ABA-inducible protein (comp79621_c0) are up-regulated by cold and saline stress. These genes are known to be induced by ABA and are involved in drought stress response in an ABA-dependent manner in other plant species (Kim, 2005). Other important transcript factors such as bHLH, NAC, MYB exhibit similar changes in expression in response to saline and cold stress.

### qPCR validation of nine genes associated with drought, cold and salinity stress response

In gene expression analysis, qRT-PCR is widely used to verify differential expression patterns measured by microarray and transcriptomic sequencing. In this study we tested the expression of five genes discussed above, including genes encoding phenylalanine ammonia-lyase and cinnamate 4-hydroxylase (Figures 5E,I) in flavonoids biosynthesis pathway, and transcription factors (TF) such as ethylene-responsive TF, NAC TF, and bZIP TF (Figures 5D,F,H). In addition, we further determined four genes that are well characterized in plant stress responses. 1-aminocyclopropane-1-carboxylate (ACC) oxidase (Figure 5A) is the last enzyme in ethylene production in plant related to ethylene regulation (Morgan and Drew, 1997; Kim et al., 1998); galactinol synthase (Figure 5B) determines the synthesis RFOs (raffinose family oligosaccharides), a group of compatible solutes in plant osmotic adjustment in drought and salinity stress (Sengupta et al., 2015); Y2K4 dehydrin variant G3 (Figure 5G) helps plants gain freezing tolerance as shown in alfalfa (Rémus-Borel et al., 2010); peroxidase (POD, Figure 5C and Supplementary Figure 1E in Data Sheet 1). Functions as the first tier of defense against reactive oxygen species (ROS) generated under drought and other stress conditions (Veljovic-Jovanovic et al., 2006). The nine genes are all up-regulated in drought, saline and cold stress responses in *O. ochrocephala*. The consistency between our qRT-PCR and the DEGs results indicate that candidate genes in stress response in *O. ochrocephala* revealed by RPKM can be a useful resource for further validation.

## Conclusion

In this study, we performed the first transcriptome sequencing analysis for a typical locoweed species, *O. ochrocephala*. This will significantly enrich available genetic information for locoweed research. In addition, by analyzing differential expression patterns of the genes under the three abiotic stress conditions, we identified multiple genes which are likely to be involved in drought, saline and cold acclimation in *O. ochrocephala*. Further, research is needed for functional analyses of these candidate genes to understand the molecular mechanism of adaptation in locoweeds to variant environments.

## Author contributions

WH and HZ designed the experiment, prepared samples for RNA-seq, analyzed the data and prepared the manuscript. YF helped in data interpretation and manuscript preparation. BG and LG analyzed the data and performed qRT-PCR analysis. XZ and LG collected plant materials. YW conceived the project, designed the experiments, supervised the analysis, and critically revised the manuscript. All authors read and approved the final manuscript.

### Conflict of interest statement

The authors declare that the research was conducted in the absence of any commercial or financial relationships that could be construed as a potential conflict of interest.

## Supplementary material

Data Sheet 1 **Supplementary tables and figures**.

Data Sheet 2 **Assembled sequences of all unigenes**.

Data Sheet 3 **Nr (non-redundant) annotation of unigenes**.

Data Sheet 4 **DEG (differential expressed genes) with annotation against Nr**.

Data Sheet 5 **GO (gene onthology) enrichment of DEGs**.

Data Sheet 6 **Hcluster heatmap with annotation and unigene IDs**.
