# Supplementary material for: De novo Transcriptome Assembly of a Chinese Locoweed (Oxytropis ochrocephala) Species Provides Insights into Genes Associated with Drought, Salinity, and Cold Tolerance
Source: Front Plant Sci. 2015 Dec 2;6:1086. doi: 10.3389/fpls.2015.01086 (PMC4667070; doi:10.3389/fpls.2015.01086)
Supplement: Data Sheet 1 — Supplementary tables and figures. [file DataSheet1.pdf]

Supplementary Table 1 Genes selected for qPCR validation and primers.

| Unigene ID | Primer set (5’ to 3’) | Length (bp) |
|---|---|---|
| comp81166_c0 | F: TGATTATTTAGAGCTTTATGGGAAG | 139 |
| | R: CCGAAACAAAGGTATTAGGTTAC | |
| comp89029_c1 | F: GAGGAGGGTGAGTGAGTAGTTG | 108 |
| | R: GCTCCCACTCCCACCTATT | |
| comp76053_c0 | F: ATAGACACTGTGGTCTCCGAG | 165 |
| | R: GGCTTACACACATAGAGCACAT | |
| comp69006_c0 | F: GGTTGTTAGGGAATCGCTTA | 170 |
| | R: GTTCCAAATAATCACTACCAAGAT | |
| comp91892_c0 | F: AACTTTAACGGTGGTTCATTC | 282 |
| | R: CATGCTATTCATCACCCAGTC | |
| comp71824_c0 | F: AAAGGGTTGGTTTATAGATTGC | 209 |
| | R: CAAACGTGATTCATGTGGC | |
| comp77463_c0 | F: TCCTGTCTTTTCTATAATTTGCTG | 177 |
| | R: GCACTACTAAATCCAAAATACAGA | |
| comp67029_c1 | F: ACACTTGGCGCATTCACG | 153 |
| | R: GCTTCCCTCAAATCTGTGCT | |
| comp76428_c0 | F: CCACCAACATTTCATTTCC | 208 |
| | R: CTTACCGAAGATTGGAACG | |
| comp67746_c0 | F: ATGGGCTTGGTGCTGTTA | 95 |
| | R:CCTTCATCAGACTAGCAGTAAACF: | |
| comp81236_c0 | F: GGGTGAATATGATGAATCTGG | 232 |
| | R: GTCTGGCTTTCAAGGACATAA | |

comp81166_c0: 1-aminocyclopropane-1-carboxylate oxidase

comp89029_c1: galactinol synthase

comp76053_c0: peroxidase

comp69006_c0: ethylene-responsive transcription factor

comp91892_c0: phenylalanine ammonia-lyase

comp71824_c0: NAC transcription factor

comp77463_c0: Y2K4 dehydrin variant G3

comp67029_c1: bZIP transcription factor

comp76428_c0: cinnamate 4-hydroxylase

comp67746_c0: *HISTONE*

comp81236_c0: *ACTIN*

Supplementary Table 2 Summary of sequencing data from different samples of treatments.

| Sample | Raw reads | Clean reads | Clean nucleotides (nt) | Q20 (%) | Q 30 (%) | GC (%) |
|---|---|---|---|---|---|---|
| CK | 58 843 192 | 55 972 722 | 5 597 272 200 | 97.62 | 93.08 | 42.79 |
| PEG | 52 514 986 | 50 079 814 | 5 007 981 400 | 97.65 | 93.14 | 42.80 |
| COLD | 65 683 442 | 62 665 844 | 6 266 584 400 | 97.58 | 92.98 | 42.89 |
| SALT | 66 708 638 | 63 491 126 | 6 349 112 600 | 97.59 | 93.04 | 42.71 |
| Total | 243 750 258 | 232 209 506 | 23 220 950 600 | | | |
| Average | | | | 97.61 | 93.06 | 42.80 |

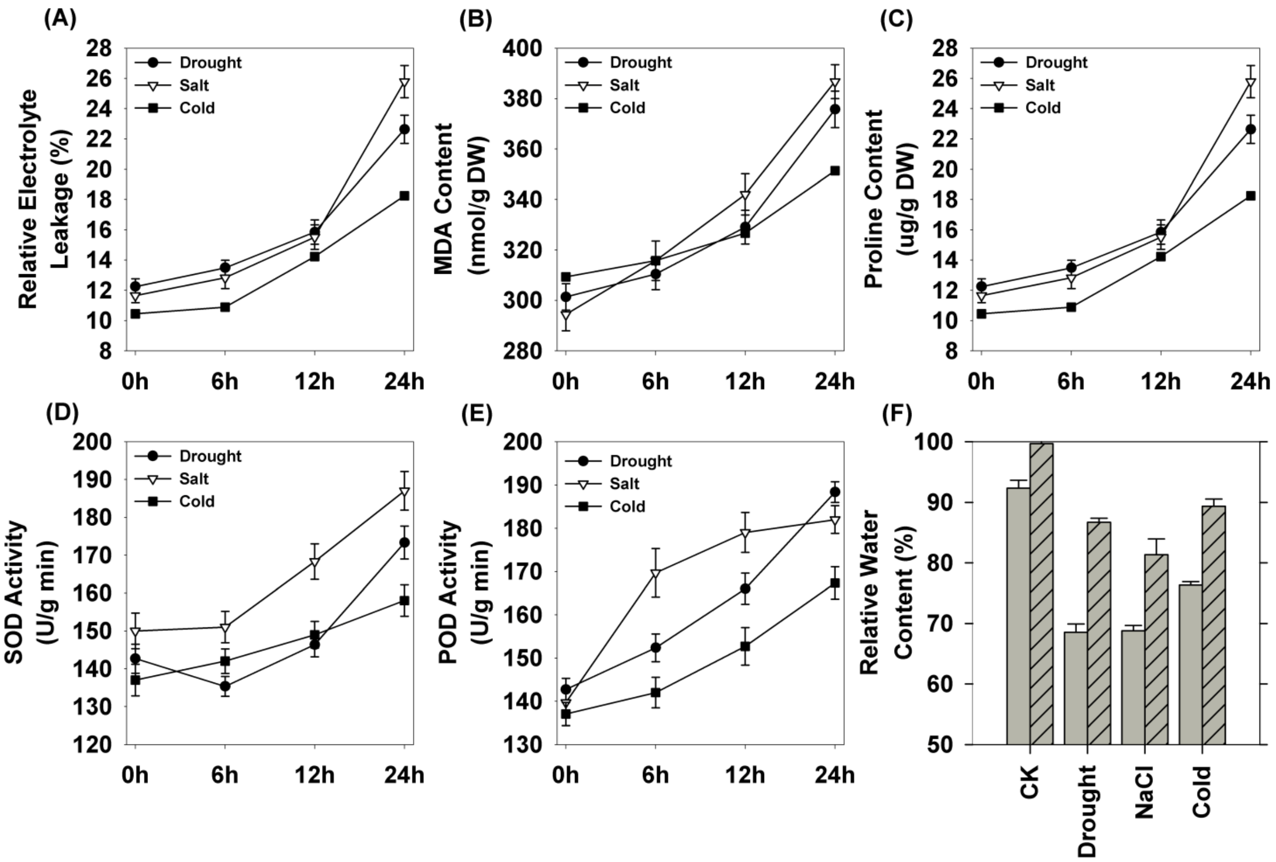


Supplementary Figure 1 Determination of seven indicators reflecting plant physiology changes under stress conditions including drought, salinity and cold. In panel (F) relative water content and survival rate were measured 96 h after the stress treatment. MDA: malonaldehyde; SOD: superoxide dismutase; POD: peroxidase. Measurement methods were according to H Z *et al.* (2015)

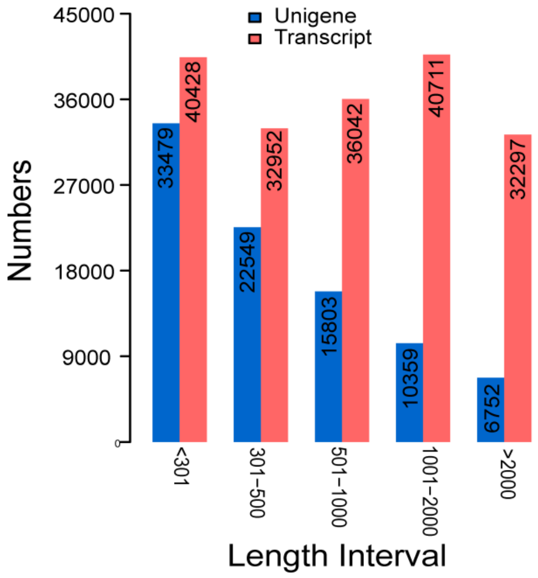


Supplementary Figure 2 Length distribution of transcripts and unigenes of the *de novo* assembly.

Length intervals (x axis) are shown for the number of nucleotides. Numbers (y axis) indicate the numbers of transcripts/unigenes for each interval.

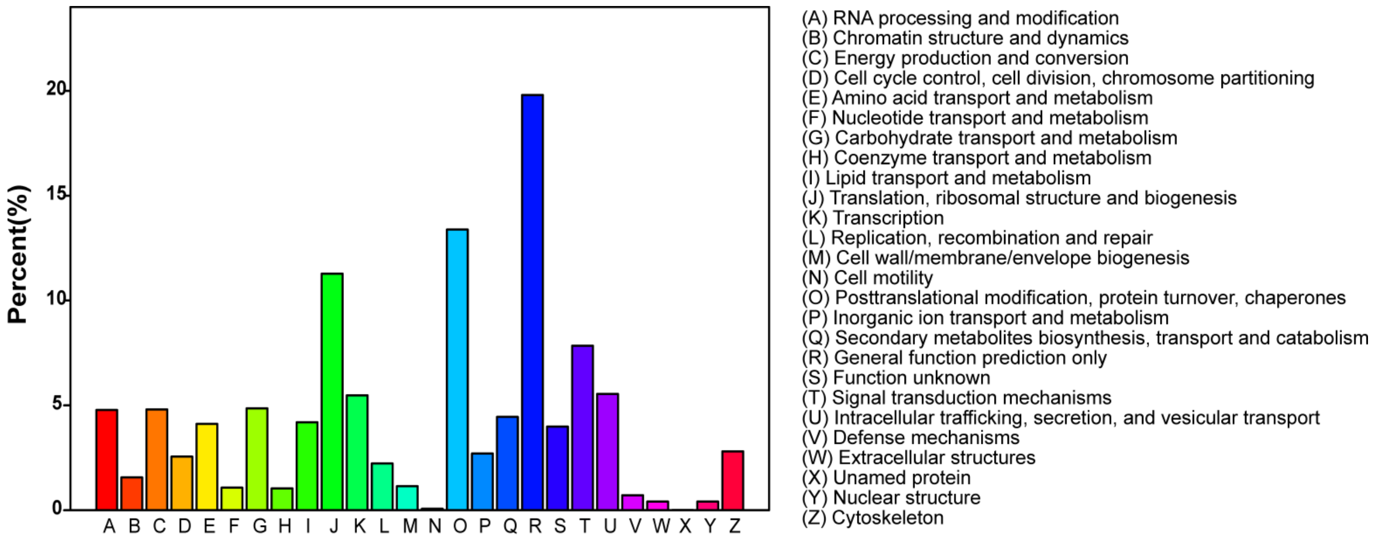


Supplementary Figure 3 KOG annotation.

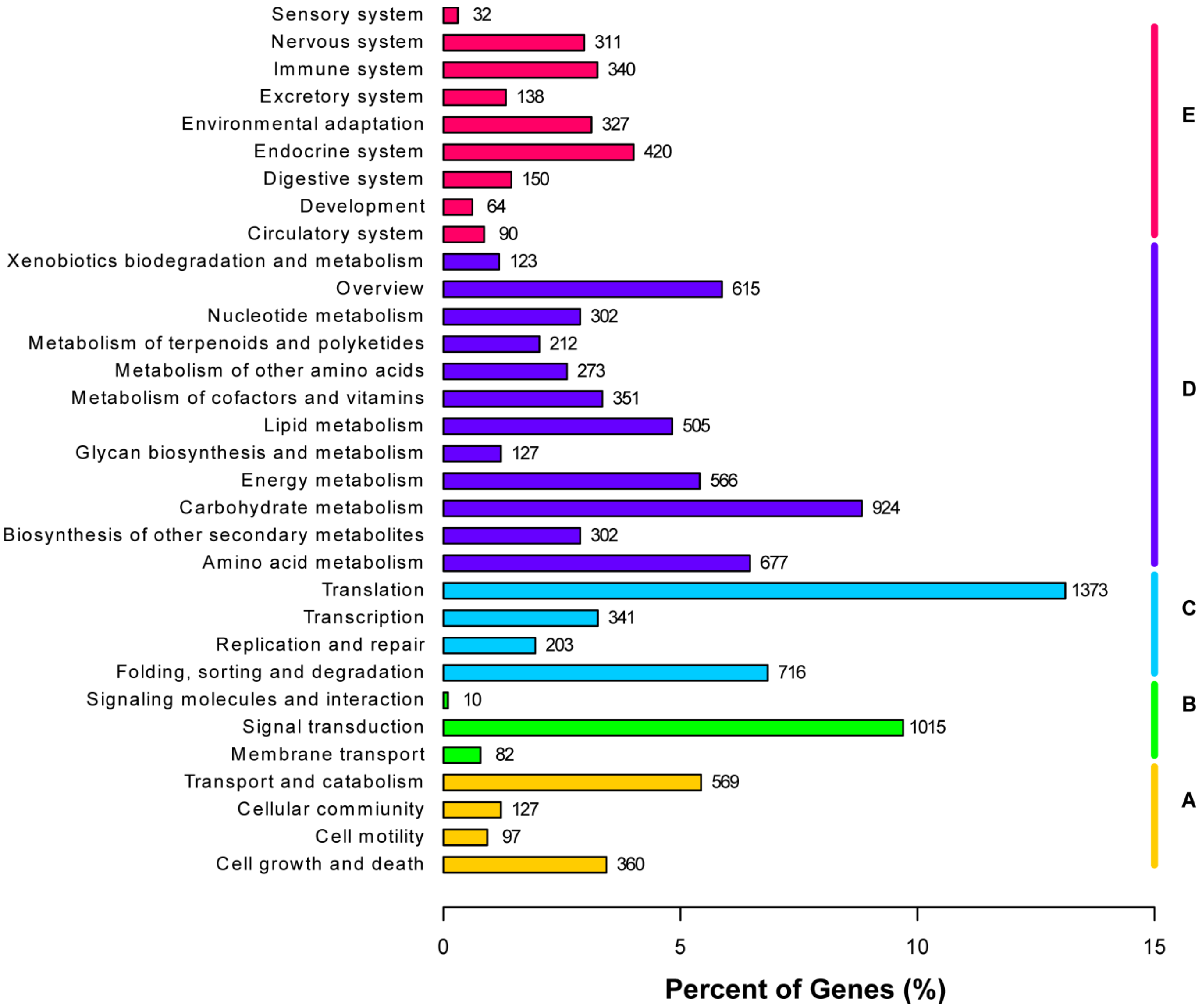


Supplementary Figure 4 KEGG annotation.

Reference

Zhuang, H., He, W., Chen, X., Wang, L., Ji, L., Guo, B., and Wei, Y. (2015). The time course of NO involved in ABA pathway to improve drought tolerance in *Oxytropis ochrocephala*. Acta Physiologiae Plantarum, 37:130
