# Supplementary material for: De novo Transcriptome Assembly of a Chinese Locoweed (Oxytropis ochrocephala) Species Provides Insights into Genes Associated with Drought, Salinity, and Cold Tolerance
Source: Front Plant Sci. 2015 Dec 2;6:1086. doi: 10.3389/fpls.2015.01086 (PMC4667070; doi:10.3389/fpls.2015.01086)
Supplement: Data Sheet 2 — Assembled sequences of all unigenes. [file DataSheet2.FASTA]

## Attention!!!

The file "data sheet 2.fasta" has been blocked. The file is larger than the configured file size limit.

URL = http://journal.frontiersin.org/file/downloadfile/102885/octet-stream/data%20sheet%202.fasta/576/1/160135
