# Supplementary figures and images for: De novo Transcriptome Assembly of a Chinese Locoweed (Oxytropis ochrocephala) Species Provides Insights into Genes Associated with Drought, Salinity, and Cold Tolerance
Source: Front Plant Sci. 2015 Dec 2;6:1086. doi: 10.3389/fpls.2015.01086 (PMC4667070; doi:10.3389/fpls.2015.01086)

## Cluster analysis of differentially expressed genes

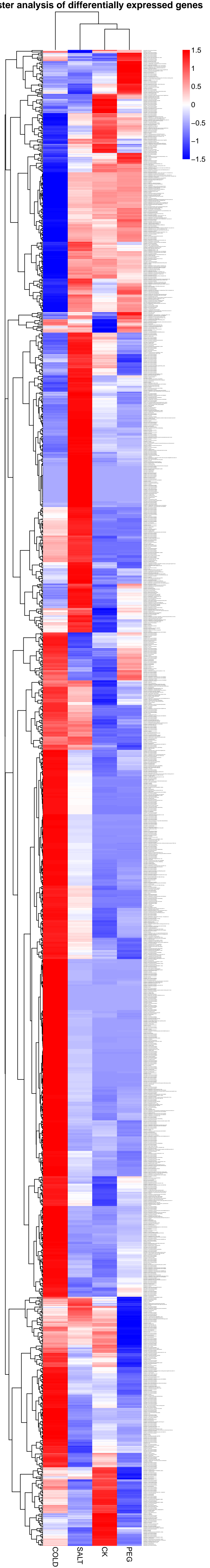

Supplement: Data Sheet 6 — Hcluster heatmap with annotation and unigene IDs. [file DataSheet6.pdf]
